# Mesh erosion into the rectum after laparoscopic posterior rectopexy: A case report

**DOI:** 10.1016/j.ijscr.2022.107136

**Published:** 2022-04-30

**Authors:** Shun Yamanaka, Tsuyoshi Enomoto, Shoko Moue, Yohei Owada, Yusuke Ohara, Tatsuya Oda

**Affiliations:** University of Tsukuba, Faculty of Medicine, Department of Gastrointestinal and Hepato-Biliary-Pancreatic Surgery, 1-1-1 Tennnodai, Tsukuba-shi, Ibaraki-ken 305-8575, Japan

**Keywords:** Case report, Rectal prolapse, Laparoscopic posterior rectopexy, Mesh erosion

## Abstract

**Introduction:**

Rectal prolapse typically presents in elderly women with protruding full-thickness rectum from the anus. Rectopexy using mesh is known to be a highly curative treatment for rectal prolapse, however, this procedure carries the risk of severe complication as mesh erosion.

**Presentation of case:**

A 78-year-old woman who had undergone laparoscopic posterior rectopexy 4 years earlier visited the outpatient clinic with a complaint of bloody stool. A colonoscopy and computed tomography revealed that part of the mesh had migrated into the rectal lumen at 8 cm from the anal verge. Based on the above findings, a diagnosis of mesh erosion into the rectum was made. Complete removal of the mesh and tacker with rectal resection was performed. Before rectopexy, the patient had severe fecal incontinence, and her anal sphincter function was decreased, therefore, Permanent colostomy was indicated instead of anastomosis. In the resected specimen, the mesh was folded and placed in the mesenteric fat of the posterior wall of the rectum, with the corner of the edge of the mesh protruding into the inside lumen.

**Discussion:**

Mesh erosion typically occurs when using mesh made of synthetic mesh and non-absorbable threads; it might induce chronic irritation and friction due to mesh shrinkage.

**Conclusion:**

To prevent mesh erosion, it is important to pay attention to the mesh materials used and ensure secure fixation.

## Highlights

• Mesh erosion into rectum after Laparoscopic posterior rectopexy was reported.
• Complete removal of the mesh and tacker with rectal resection was needed.
• Colostomy was made because of existence of severe fecal incontinence, preoperatively.
• Paying attention to the Shrinkage and secure fixation of synthetic mesh.

## 1 Introduction

Rectal prolapse is a common disease that typically presents in elderly women with protruding full-thickness rectum from the anus. Rectal prolapse is attributed to frail pelvic floor muscles accompanied by aging and childbirth, and it causes severe discomfort around the perineum. Surgical intervention is sometimes needed for treatment [1].

There are two procedures for surgically managing rectal prolapse: trans-abdominal approach and the trans-perineal approach. In elderly patients, the operative procedure is determined while taking into account the extent of prolapse, surgical invasiveness and expected quality of life after the operation [2].

The trans-abdominal approach is a more radical method of curing rectal prolapse and has a better surgical outcome than the trans-perineal approach. Mesh is used for rectopexy in the trans-abdominal approach to fix the rectum to the sacrum. However, this mesh is associated with specific complications, especially rare but severe complications of rectal erosion, which are observed in 1.7% of cases [3].

We herein report a case of mesh erosion into the rectum after laparoscopic posterior rectopexy along with a literature review. This report is in line with the SCARE criteria [4].

## 2 Case report

**Fig. 1:**
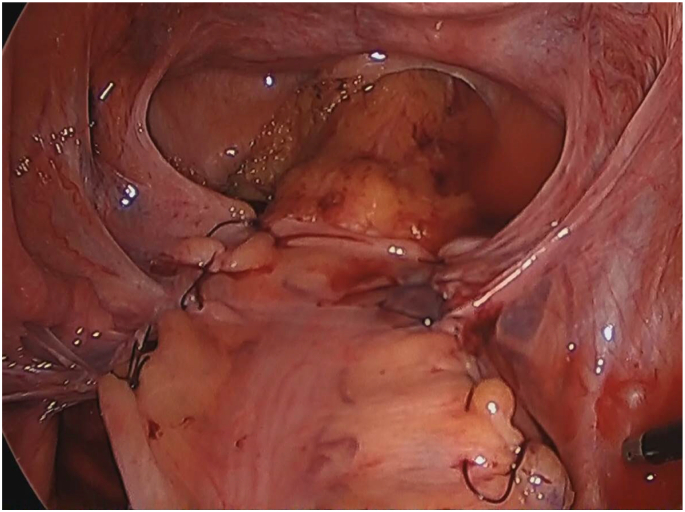
Intra-abdominal findings after laparoscopic rectopexy (Well's procedure). The mesh was fixed firmly to the rectum and sacrum, and the whole mesh was completely covered with peritoneum.

A healthy 78-year-old woman had been suffering from 10-cm rectal prolapse from the anus for several years. The patient had undergone laparoscopic posterior rectopexy via the Wells technique four years ago. At the operation, synthetic mesh composed of polypropylene at the sacral attachment side and expanded polytetrafluoro-ethylene at the organ attachment side had been used to fix the rectum. After the rectum was mobilized from the promontory of the sacrum down to the pelvic floor, the mesh was trimmed to three-quarters of the circumference of the rectal wall and then placed at the dorsal side of the rectum, where it was fixed with tackers at the sacrum. The mesh was then sutured and fixed to the rectal wall with absorbable string, and the whole mesh was completely covered with peritoneum (Fig. 1). The patient was followed up in the outpatient clinic without any symptoms, including recurrence of rectal prolapse.

**Fig. 2:**
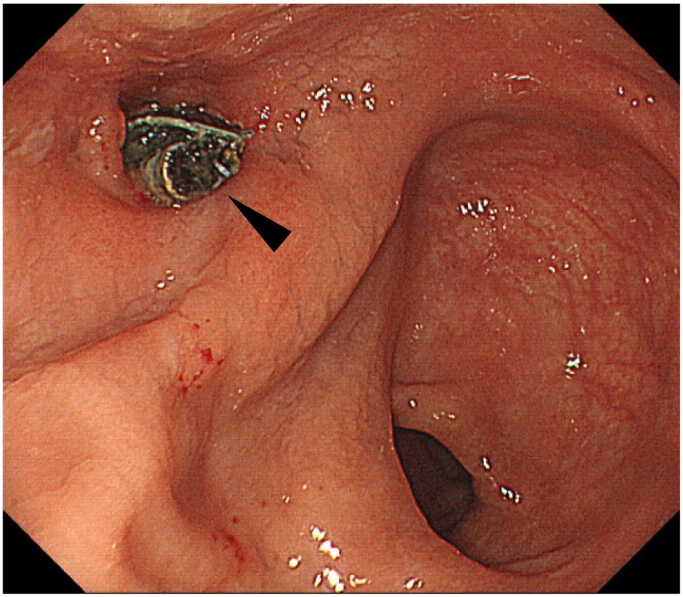
A colonoscopic examination revealed that part of the mesh had migrated into the rectal lumen 8 cm from the anal verge (black arrowhead).

**Fig. 3:**
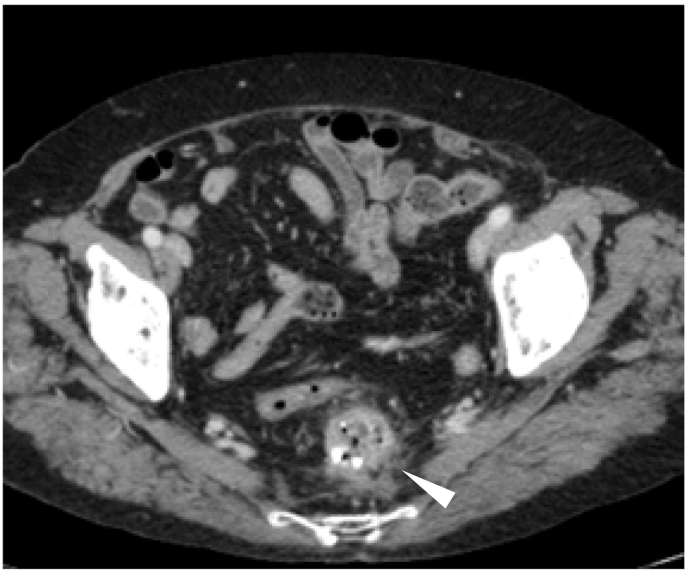
Computed tomography showed a diverticulum-like structure with panniculitis in the fat tissue behind the middle rectum, and a high-density structure was identified inside the diverticulum-like structure (white arrowhead).

Four years after the surgery, the patient complained of a small amount of bloody stool. A colonoscopy examination revealed that part of the mesh had migrated into the rectal lumen at 8 cm from the anal verge (Fig. 2). Computed tomography showed a diverticulum-like structure with panniculitis in the fat tissue behind the middle rectum, and a high-density structure was identified inside the diverticulum-like structure (Fig. 3). Based on the above findings, a diagnosis of mesh migration into the rectum was made.

**Fig. 4:**
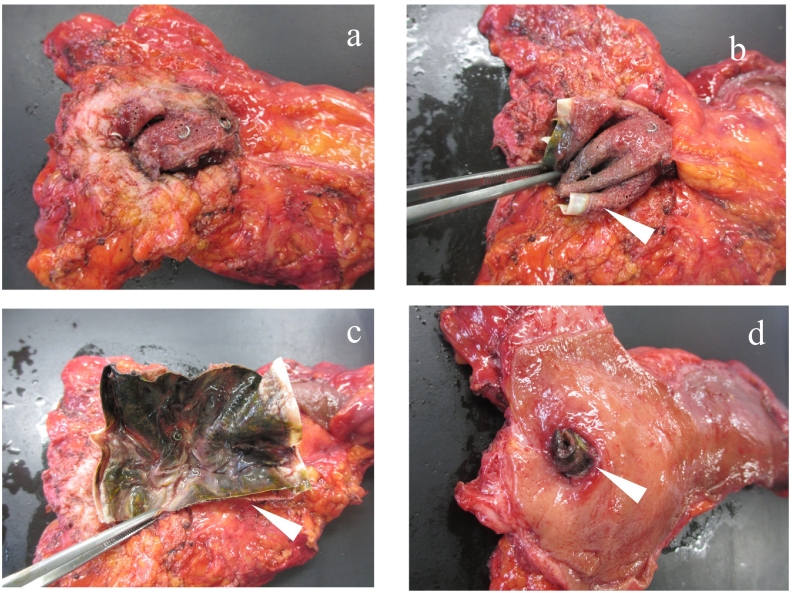
Rectum was resected with the mesh and tacker (panel a), the mesh (white arrow head) was folded like a mesh plug for inguinal hernia repair and went into the mesenteric fat of the posterior wall of the rectum (panels b, c), and the corner of the edge of the mesh protruded into the inside lumen (panel d).

Complete removal of the mesh and tacker with rectal resection was performed by experienced colorectal surgeon. Before rectopexy, the patient had severe fecal incontinence and her anal sphincter function was decreased, therefore, Hartmann's operation was indicated instead of anastomosis. Intraoperative findings showed that the posterior wall of the rectum, the anterior surface of the sacrum and the mesh were tightly adherent, with firm scars. The mesh and tacker were completely removed from the presacral scar tissue by sharp dissection with electric scissors. The rectum was resected with the mesh and tacker, and sigmoid colostomy was constructed. In the resected specimen, the mesh was folded like a mesh plug for inguinal hernia repair and placed in the mesenteric fat of the posterior wall of the rectum, with the corner of the edge of the mesh protruding into the inside lumen (Fig. 4a, b).

The patient had a good postoperative course without any complications except for postoperative dysuria. She is still followed up in the outpatient clinic without any additional interventions.

## 3 Discussion

Various surgical procedures are available for managing rectal prolapse, being mainly categorized into trans-abdominal and trans-anal approaches. Transabdominal rectopexy might be recommended for the treatment of rectal prolapse if a patient is deemed able to tolerate such an operation [5]. Regarding the postoperative recurrence of prolapse, trans-perineal surgery, represented by the Delorme's operation and Altemeier's operation, has a high recurrence rate of 26.5% compared with trans-abdominal approach of 5.2% [6].

Three procedures of trans-abdominal rectopexy have been performed: anterior sling rectopexy (the Ripstein method), posterior rectopexy (the Wells method) and ventral rectopexy. These procedures use mesh to fix the rectum to the sacrum, and tackers or absorbable/non-absorbable threads are used to connect the mesh to the organs. Posterior rectopexy and Ventral rectopexy generally results in a better outcome in terms of recurrence and quality of life after the operation [5], [7], [8]. The Ripstein operation is being used less and less due to severe postoperative morbidity and dyspepsia in 13% of patients postoperatively [9]. Due to low recurrence rates, good functional results, and low rate of mesh complications, laparoscopic and robotic rectopexy increased popularity and current standard for treatment of rectal prolapse [10]. However, the meta-analysis showed robotic assisted rectopexy may ensure limited improvements in post-operative outcome if compared to laparoscopic operation. RCTs are needed to compare both procedure [11].

Representative major complications after trans-abdominal rectopexy are ileus (3%), wound infection (3%), and scar hernia (1%) [12]. Rectal mesh erosion is a rare but severe complication after trans abdominal rectopexy, occurring in less than 2% of cases [3].

Mesh erosion is phenomenon whereby rectum wall becomes damaged as a result of chronic contact and mechanical irritation or friction with implanted mesh for a long period [13].

The frequency of mesh erosion differs between synthetic and biomaterial meshes, with reported rates of 7–14% and 0%, respectively [14]. In synthetic mesh, the two most widely implanted grafts were polypropylene and polyester mesh, but polyester mesh gives a higher risk of mesh exposure compared polypropylene implants [15]. Implantation of mesh induces inflammation and subsequently contraction and shrinkage. Shrinking of the mesh induce mesh exposure to organs and making a space between rectum and sacrum and exposed mesh may have a greater chance of mechanically irritating the rectum than biological materials. Both mesh showed shrinkage with 46% and 26%, respectively, after follow up of 18 month [16].

Using non-absorbable thread for mesh fixation can also cause mechanical irritation and may induce mesh erosion. There are also differences in the frequency of mesh erosion between absorbable and non-absorbable thread which is used for mesh fixation. The erosion occurred at a frequency of 2% in the nonabsorbable suture group, including erosions into the rectum and the vagina. On the contrary, there was no erosion in the absorbable suture group [17].

According to Nilesh et al., deeper stitches through the rectal wall and ischemia secondary to excessive tension placed around the rectum are both risk factors of mesh erosion. Therefore, it is important not to stitch the needle too deeply to the rectal wall when suturing the mesh, and to adjust the position of the stitch not to make an excessive tension on the rectal wall [18]. To prevent the mesh erosion, it may important not to stitch the needle to the rectal wall that is too distant from sacrum so that there is no excessive tension on the rectal wall.

Treatment of mesh erosion depends on the location of erosion, severity of mesh protrusion into the rectum, presence of infection, and degree of fibrosis around the area of mesh. Mesh erosion is typically treated with surgery including trans abdominal or trans anal mesh removal and involved tissues in the fashion of rectal resection with or without stoma formation [19]. In recently, organ preserving techniques in the management of mesh erosion was reported. Partial or complete mesh resection by using trans anal endoscopic surgery or transanal minimally invasive surgery without rectal resection, combined with laparoscopic pelvic assessment and detachment of mesh from the sacral promontory [20]. Despite of requiring multiple procedure and months to complete, this technique would be available for the elderly patients with mesh erosion who may not wish to have a rectal resection.

In the present case, synthetic mesh was used, with absorbable thread and tacker was used for fixation to sacrum. This may have caused the mesh to move, potentially contributing to its erosion into the rectum. Rectal resection was needed because of preoperative severe fecal incontinence.

## 4 Conclusion

Postoperative mesh erosion into the rectum is a rare but severe complication after rectopexy. The main reason for this event is considered to be continuous mechanical irritation between the mesh and rectum under conditions of continuous peristaltic movement. Mesh made of synthetic materials and non-absorbable threads might induce chronic irritation and friction due to mesh shrinkage. To prevent mesh erosion, it is important to pay attention to the mesh materials used and ensure secure fixation.

## Source of funding

This research did not receive any specific grant from funding agencies in the public, commercial, or not-for-profit sectors.

## Ethical approval

Not applicable.

## Consent for publication

Written informed consent was obtained from the patient and her family for the publication of this case report and the accompanying images. A copy of the written consent is available for review by the Editor in Chief of this journal upon request.

## Research registration

Not applicable.

## Guarantor

Tsuyoshi Enomoto.

## Provenance and peer review

Not commissioned, externally peer-reviewed.

## CRediT authorship contribution statement

SY contributed to play important role in clinical setting and wrote of the manuscript. TE is the corresponding author and conceptualization of treatment and the manuscript. YO, YO, YA, TO revised and approved the final manuscript.

## Declaration of competing interest

All authors declare no conflicts of interest.
